# Systematic characterization of faecal sludge from various sources for its use as a solid fuel

**DOI:** 10.1007/s13399-023-04906-2

**Published:** 2023-09-23

**Authors:** Niharika Sharma, Berend Lolkema, Peter Mawioo, Christine Maria Hooijmans, Capucine Dupont

**Affiliations:** 1 Department of Water Supply, Sanitation and Environmental Engineering, IHE Delft Institute for Water Education, Westvest 7, 2611 AX Delft, the Netherlands; 2 University of Eldoret, Eldoret, Kenya

**Keywords:** Solid fuel, Faecal sludge, Co-combustion, Higher heating value, On-site sanitation systems, Elemental analysis

## Abstract

Faecal sludge (FS) is not extensively evaluated for its potential as a solid fuel mainly due to the general conception of its “highly variable characteristics” in relation to the wide range of on-site sanitation systems. An extensive and systematic FS characterization was therefore conducted on twenty-four samples collected directly from pit latrines, ventilated improved pit latrines (VIPs) and urine-diverting dehydrating toilets (UDDTs) at two depths to understand the impact on properties relevant for combustion. The higher heating value (HHV) for these samples lies between 13 to 22 MJ/kg DM (dry matter). However, such high values should be taken with caution since the measurement guidelines recommended the removal of the large inert pieces found in FS. Besides this potential bias of procedure, differences could be observed between containments, where pit latrines showed the largest variability as compared to VIP and UDDT. These differences are mainly correlated with the ash content, ranging from 15 to 50% DM, while the organic elements concentrations were similar for all samples. Interestingly, the same major inorganic elements could be measured in all samples, namely Si, P, Ca and K followed by Mg and Na. Such similar composition is probably due to similar sanitation practices and staple diet. However, the overall concentration of minor elements was below 1000 ppm DM for most samples. The N concentrations were quite high, between 2.5 to 4.5% DM. Abovementioned results may be problematic for process and environmental aspects if FS is combusted alone. FS can therefore be suitable preferably for co-combustion in blend with lignocellulosic biomass waste.

## Introduction

According to Berendes, Sumner [1] and Strande and Brdjanovic [2], globally, about 2.7 billion people depend on on-site sanitation systems (OSS) in rural and in urban areas, and this number will likely increase as a result of the expected increase in the global population from 7.5 billion in 2017 to 9.7 billion in 2050 [3]. Faecal sludge (FS) that accumulates in OSS is very often not managed safely, contaminating the environment and impacting public health severely [2, 4]. Exploring the potential of FS as a fuel can provide financial assistance for improving planning and management across the sanitation value chain [2]. However, FS is not extensively evaluated for its potential as a solid fuel, mainly due to the general conception of “highly variable characteristics” due to the wide range of on-site containment systems used in OSS [5, 6].

The key properties to identify the performance of solid fuel encompass moisture content, calorific value or higher heating value (HHV), ash content and organic and inorganic elemental composition [7–9].

The number of studies on FS characterization for use as a solid fuel is limited (around 20). As described in the studies, samples were collected in cities from different countries worldwide and containment types. Bakare and Foxon [10], discussed the heterogeneity within and across VIPs, with sampling performed at different depths of 0m, 0.5m, 1.0m and 1.5m. Nearly all studies were limited to a few samples in total, typically around 5 to 10 samples, and only one recent study considers 30 samples, which makes statistical evaluation possible [11]. In terms of properties, it is noteworthy that due to practical reasons, most studies were carried out on dry samples. Hence, they did not mention the moisture content, or the total solids. Only Krueger measured it and discussed the dewatering aspect [11].

The studies usually focused on HHV, for which a large range of values could be observed on dry basis, from 5 to 24 MJ/kg DM [12]. Prior studies reported the addition of inorganic compounds in the OSS, like sand, which in turn decreased the HHV, or the addition of organics like wood chips, oil and sawdust, which in turn increased the HHV of FS [13].

Around 10 studies did measurements on organic elemental composition, i.e. C, H, N, S and O [6], O being obtained by difference as nearly always the case in solid characterization. Again large differences could be observed between the studies, with values for C on dry basis ranging from 27% DM [5] to more than 40% DM [11]. Such differences can be linked to the ash content and one can expect much more similar values if the measurements are expressed on dry-ash-free basis. Few literatures tied the increase in FS ash fraction with the digestion of its organic matter which decreases the C, H and O fraction via CH_4_ and CO_2_ release [12, 14]. Muspratt and Nakato [14] thoroughly examined the decrease in HHV by 25–40% with the increase in digestion of FS on drying beds. Infiltration of sand or soil in FS also contributes to the increase in ash content [12]. Seck and Gold [15] and Ward and Gold [16] observed an increase in ash content of 6% and 20% for FS dried on sand drying beds.

Volatile solids (VS) and ash content, which are the measure of the organic and inorganic matter, have also been determined quite extensively, since it constitutes a basic FS analysis, even when the use as solid fuel is not targeted. The recent extensive study done by Krueger and Fowler [11] reported the ash content for UDDTs and VIPs in the range of 40–61% DM and 18–81% DM respectively. He explained that the high variation in VS and ash content could be due to varied FS collection techniques and the type of containment.

However, the information is limited on ash composition and more generally on the inorganic elements in FS. As N and S are measured using the same device as C and H, they were reported in around 10 studies too. Werther and Ogada [17] and Roy and Dutta [18], compared dried FS with the traditional fuels like coal and reported comparatively higher concentrations of N, S and Cl and the potential to form harmful species during combustion, such as dioxins, furans, NO_x_, N_2_O, SO_2_ and HCl. Few articles reported the data on Cl and the heavy metals [5], notably focusing on their potential environmental impact through gas emissions during combustion or if FS ash is disposed in the soil—studies have also measured heavy metals in FS without fuel as target, since it is important to evaluate FS toxicity.

Hardly any research was done on the major inorganic elements contained in FS, such as K, Ca, Si, Na or P, although those are known to be strongly influential in combustion units, since they can lead to ash fouling or slagging, and thus failure of the reactor [19]. Only three recent studies could be found [5, 11, 20]. Gold and Ddiba [5] results should be taken with some caution since they made measurements via X-ray fluorescence, which is not the standard method used for solid characterization and subject to discussion in terms of reliability, as discussed previously [9]. Krueger and Fowler [11] did not discuss the impact on combustion behavior, and surprisingly did not report any Na. Although Krueger and Fowler [11] collected thirty samples, it highlights the criticality of sampling protocol which when differs could give altered data. Furthermore, the high K and Na led them to the conclusion that FS cannot be used alone as solid fuel and needs to be mixed with samples poorer in these elements to avoid ash problems.

Although few studies have successfully justified the worthiness of FS as a solid fuel, the explicit impact of FS properties on the fuel potential is still unclear. This includes the lack of information on the explicit impact of the type of OSS containment and possible heterogeneity associated with it. In addition, the data from previous researches are drastically scattered because of lack of dedicated standards methods available for FS collection and analysis. Thus, researchers improvise the methods based on their requirements, which makes it hazardous for data comparison.

To contribute to fill in the existing knowledge gap associated with inconsistency, this research systematically characterized twenty-four FS samples collected from three on-site containment systems in Kenya, namely pit latrines, ventilated improved pit latrines (VIP) and urine-diverting dehydrating toilets (UDDT) at two different depths namely top (T) and middle (M). The study investigated the physico-chemical properties of the collected FS in terms of properties relevant for combustion processes, including HHV, ash content, organic and inorganic elemental composition, following the standard methods recommended on dry basis [6], subsequently creating a database which evaluated the heterogeneity associated with FS and its suitability to use it as a solid fuel comparing it with different feedstock.

Please note the dataset generated during the research will be available from the corresponding author upon request.

## Methodology

### Sample collection and preparation

**Fig. 1:**
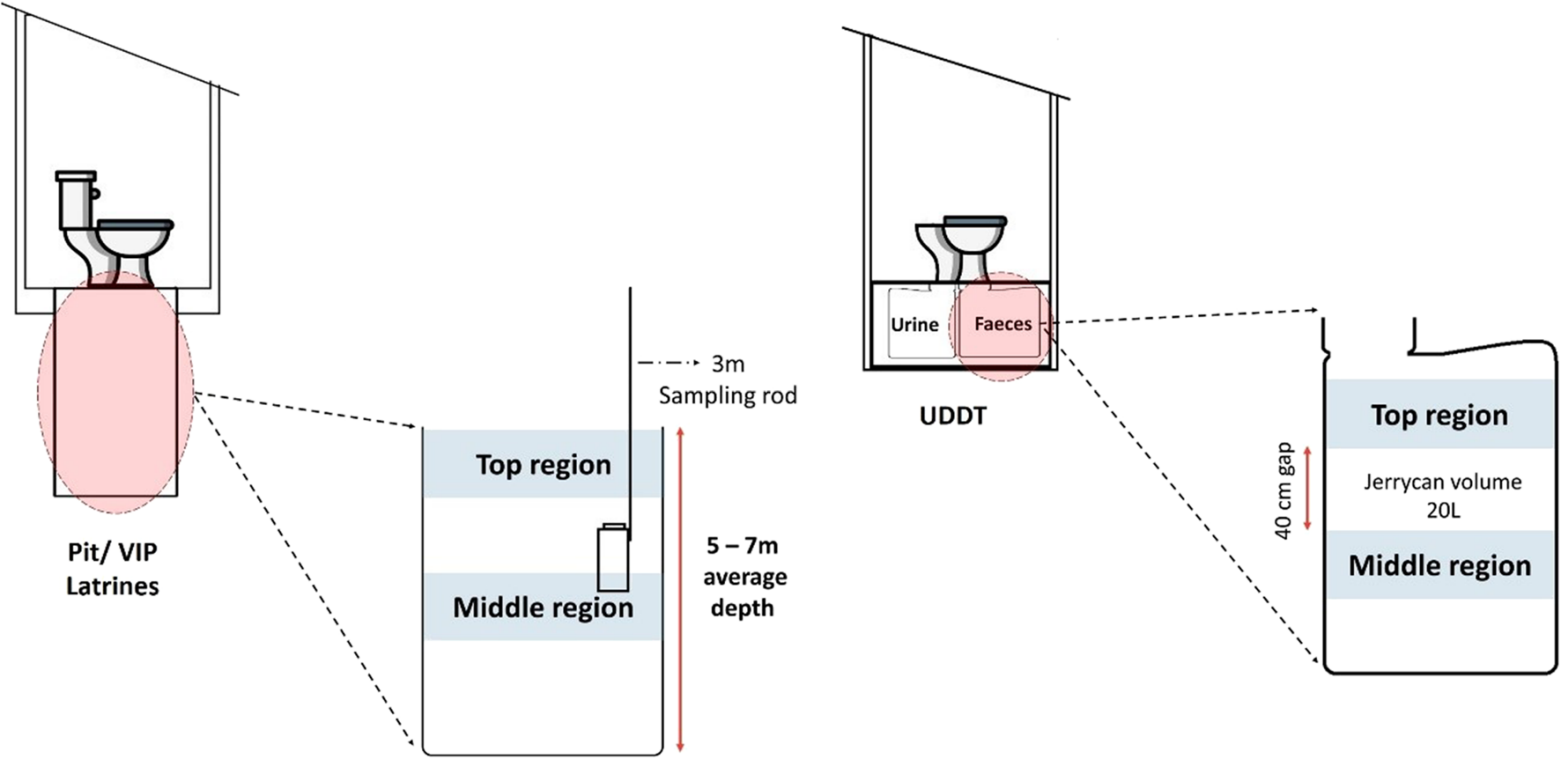
Two sections at which FS samples were collected from pit, VIP latrines and UDDTs

This study was performed using FS samples collected from three types of OSS containment facilities distributed over twelve randomly selected sites in Naivasha, Kenya, including five pit latrines, three VIPs and four UDDTs. Samples were taken from each containment from two different depths, with a 3-m difference for pit and VIP latrines and 40 cm for UDDTs, namely top (T) and middle (M), as shown in Fig. 1.

The summary of sample distribution based on the location and type of containment is shown in Table 1.

**Table 1:** Sample breakdown with respect to containment type and location

OSS type	Location	No. of containments	Total samples
Pit latrine	Maryland Kayole Mirera Old Station	1 2 2	10
VIP latrine	Maryland Kayole Old Station	2 1	6
UDDT	Karagita Kihoto NTP^1^ site male NTP^1^ site female	1 1 1 1	8

^1^*NTP* National Target Population site (male and female)

In Naivasha (Kenya), the samples were collected and dried at 105°C. Before drying the samples, initial sorting was performed by the sampling team to remove non-faecal material like large pieces of glass, fabrics etc. However, the exact removed quantity of non-faecal material was not recorded. The field team also gathered supplementary information concerning general sanitation practices, such as the lining of the pits, anal cleansing material, solid waste disposal practices if any, connection to the bathroom, among others which were tracked with experimental results for behaviour interpretation of FS samples.

The collected samples were shipped to IHE Delft Institute for Water Education, the Netherlands, for laboratory analysis. At IHE Delft, sub-sampling was done using the quarter split method [21] and samples were grinded and homogenized using an electric blender *Bamix DeLuxe L160*. In addition, larger non-faecal matter (or solid waste) like stones, gravels, fibre, plastic pieces, glass pieces and paper, among others, were removed before grinding the samples following “Methods for Faecal Sludge Analysis” [6]. Hereafter, the samples were analyzed in *triplicates*.

### Ash content

The ash content (also known as fixed solids) in % dry matter (DM) was determined following *Method 2540E* [22]. A known sample was ignited in a muffle furnace at 550°C until a constant weight was achieved, and using initial and final weights, the ash and VS were calculated. Since the samples were pre-dried before shipping, determining moisture content or total solids could not be done.

### Higher heating value

The calorific value was measured in terms of higher heating value (HHV) in MJ/kg DM using a Parr 6200 Bomb Calorimeter following standard from ASTM D5865 [23]. The grounded samples were dried at 105°C prior to the analysis to ensure complete dryness. The procedure was slightly adapted as the samples were not sieved with a 250-μm sieve and the samples which were not pelletable were analyzed in powder form to avoid the addition of any binder [6].

### Organic elemental composition

The organic elemental composition (C, H, N, S, O) was performed in collaboration with an external laboratory (Sophisticated Analytical Instruments Laboratories, Thapar Technology Campus, Thapar Institute of Engineering and Technology, Patiala, Punjab, India). *Flash 2000 by Thermo fisher Scientific* was used to perform the ultimate analysis with thermal conductivity detector under He and O_2_ with purity 99.995% [24] adapting from ASTM D3176-89 [25]. Calibration of the instrument was carried out using the standards *Sulphanilamide* (4-amino benzene sulphonamide) and *BBOT* [2,5-bis(5-tert-butyl-2-benzo-oxazol-2-yl)]. To determine the elemental constituents, C was converted to CO_2_, H to H_2_O, N to N_2_ gas or oxides of nitrogen. The samples were tested in duplicate and the final values were recorded in terms of % DM. The O content was calculated by difference using Eq. 1 [25].

1 $$O\left(\%\right)=\left[100-\left(\%C+\%H+\%N+\%S\ \right)\right]-\% ASH$$

### Inorganic elemental composition

To determine inorganic composition U.S. EPA [26], United States Environmental Protection Agency [27] was followed. 0.4 g of sample was digested using concentrated HNO_3_ and *BCR-146 Sewage Sludge* was used as a reference material following ASTM D5198-17 [28]. The BCR-146 recovery percentage was determined at the end to validate the concentration for metal concentration in samples [29]. Once the digestion was complete, the samples were first transferred to a known volume of 50 mL followed by 4 times dilution to analyze in *PerkinElmer Inductively Coupled Plasma-Optical Emission Spectrometry* (ICP-OES). The analyzed elements were potassium (K), sodium (Na), calcium (Ca), magnesium (Mg), iron (Fe) and zinc (Zn).

To determine the phosphorous (P), around 0.3–0.4 g of dried FS samples were destructed by H_2_SO_4_–Se digestion mixture and placed in oven at 100°C for at least 2 h. Finally, around 10–12 ml of H_2_O_2_ was slowly added followed by placing the tubes in pre-heated block at 300°C until digests turned colorless or light-yellow [30]. BCR-129 or *Hay Powder* was used as a reference sample during the analysis [31]. The digests were then transferred to known volume and with suitable dilution, P was estimated using *ascorbic acid method* or *Method 4500-PE* [22] against 880-nm wavelength in PerkinElmer spectrophotometer [32].

Lastly, silica (Si) content in FS sample was analyzed using the colorimetry analysis following ASTM E463-21 [33]. The FS samples were first ashed following Method 2540E [22] until constant weight. Known weight of 50 mg of ash was melted in excess of NaOH (around1.5 g) at 350°C in a muffle oven followed by neutralization using 1:1 HCL solution and diluting to 1L digested solution. Then known volume of solution was reacted with ammonium molybdate to form silica molybdic acid (H_8_Si(Mo_2_O_7_)_6_), which produces an intense yellow color which was further neutralized by the tartaric acid and converted to molybdenum complex by means of 1-amino-2-naphthol-4-sulfonic acid and sodium hydrogen sulfite solution. The color developed is proportional to Si content and the intensity was measured corresponding to 650-nm wavelength in spectrophotometry.

## Results and discussion

### Ash content

**Fig. 2:**
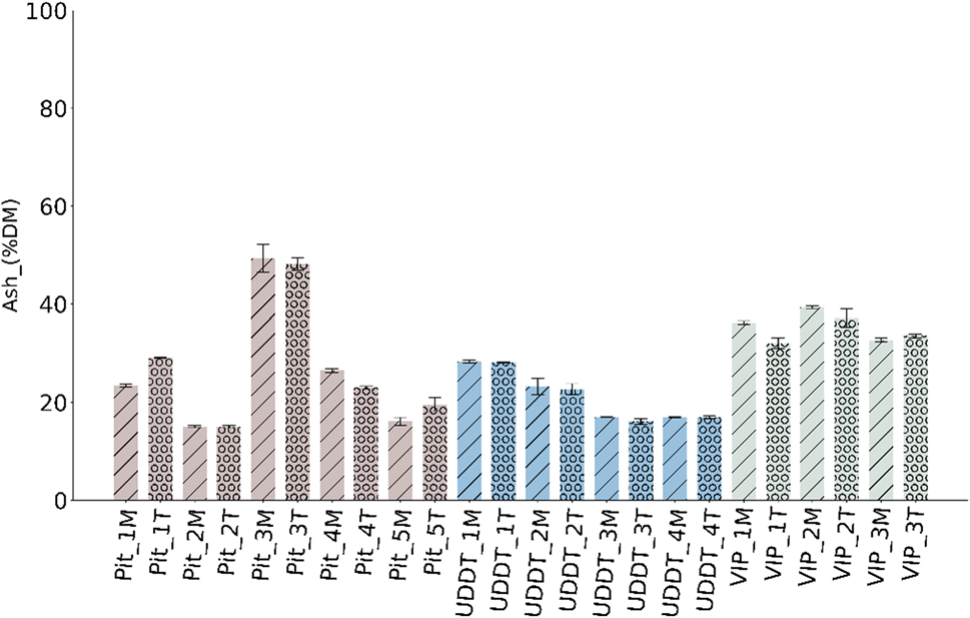
Ash content of FS in % DM

As depicted in Fig. 2, the ash content ranged between 15 and 30% DM within UDDT and pit latrine, with an exception of Pit_3 with 50% DM. It was conveyed by the sampling team; in order to contain the odor from these toilets, lime was added. Thus, such eccentricity in ash content in Pit_3 can be attributed to the addition of lime. The ash content from VIPs showed a broad range of 30–40% DM. No clear influence on ash content from the depth in containment could be observed, in agreement with [11]. On contrary, Zziwa and Nabulime [34] and Zuma and Velkushanova [35] reported an increase of ash content with increase in depth 1.5 m. Such increase would appear logical, since the longer the FS is in the containment, the higher the decrease of VS due to anaerobic digestion or stabilization [12, 36, 37]. More analysis and data with regard to the depth of containment could aid in well-grounded trend between the two.

The high variability in ash content within pit latrines is assumed to be due to the percolation of liquid (water) into the soil, as in this case these unlined pit latrines and VIPs. As a consequence, at the time of emptying the containment or during sample collection, soil gets mixed with FS which in turn increases its concentration. In addition, the OSS are often used for solid waste disposal of menstrual hygiene products waste, condoms, lime, batteries, textiles, food waste etc. It was inevitable during this research as reported by the field team. The ash in case of the pit latrines and VIPs is thus thought to be more due to external pollution sources than intrinsic to the FS. Such a high ash content was also previously reported and discussed in [10, 12, 14, 15, 20, 35]. In UDDTs, ash content is relatively less high < 30% DM, as the sludge is collected in separate containers and emptied every 2 days, as reported by the field team.

When comparing these ash values with those of lignocellulosic biomass, FS clearly has much higher ash content, where ash content of majority of the lignocellulosic biomass is less than 10% DM [38, 39]. While there is no strict threshold for the ash concentration in combustion, high values are problematic, since high amounts of residues can be expected at the end of combustion. In addition, slagging, fouling or agglomeration can happen in the combustion reactor, depending on the inorganic elements constituting this ash. Thus, serious thought should be put when considering waste like FS with such high ash content towards efficient ash removal mechanisms during and after combustion [38, 40].

### Composition in C H N S

**Fig. 3:**
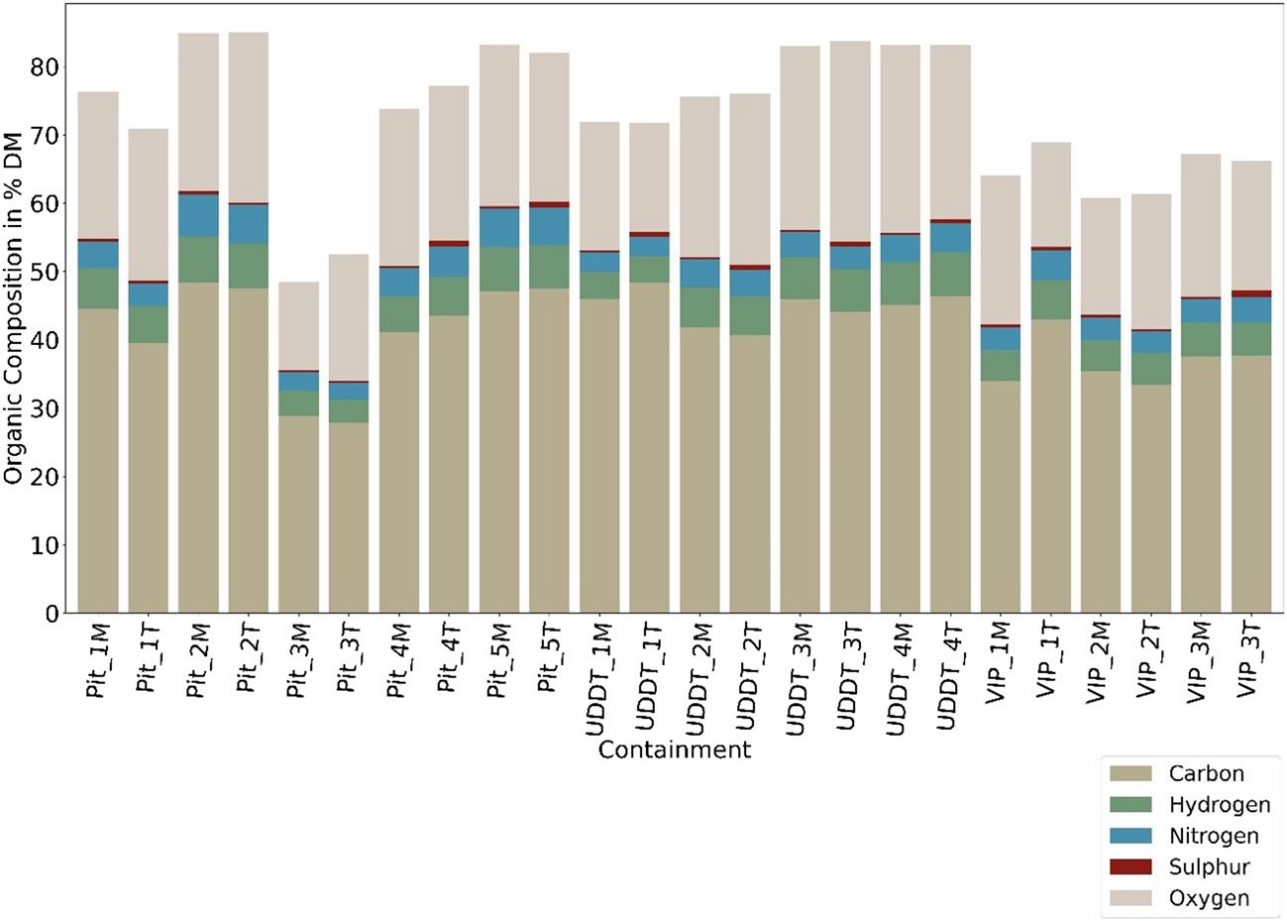
Organic elemental composition of FS in % DM

Fig. 3 shows the organic concentration of FS in terms of C (28–48% DM), H (3–7% DM), N (3–6% DM) and S (< 1% DM). The highest variability was primarily observed for C within the pit latrines. The lowest value was in the range of 28% DM for Pit_3, corresponding to the highest ash content of about 49% DM. The low C content in Pit_3 is attributed to an excess concentration of inorganics in the pit latrine due to the addition of lime (to contain the odor from FS, as explained above) which increased the inorganic content by further stabilizing the FS [12], whereas the highest C was observed in the range 47–48% DM for Pit_2. Moreover, the C content across UDDTs are higher as compared to VIPs and pits with lower variations and deviations observed than that of VIPs with a range of 34–43% DM. Correspondingly, for H and N fractions, the range was 4–6% DM and 3–6% DM, the lowest being in Pit_3 (3.4–3.7% DM), again most likely to be caused by the addition of lime. For UDDT_1, the H and N were comparatively lower (3.9–4.1% DM) than other UDDTs. No proper explanations could be derived for such trend.

**Fig. 4:**
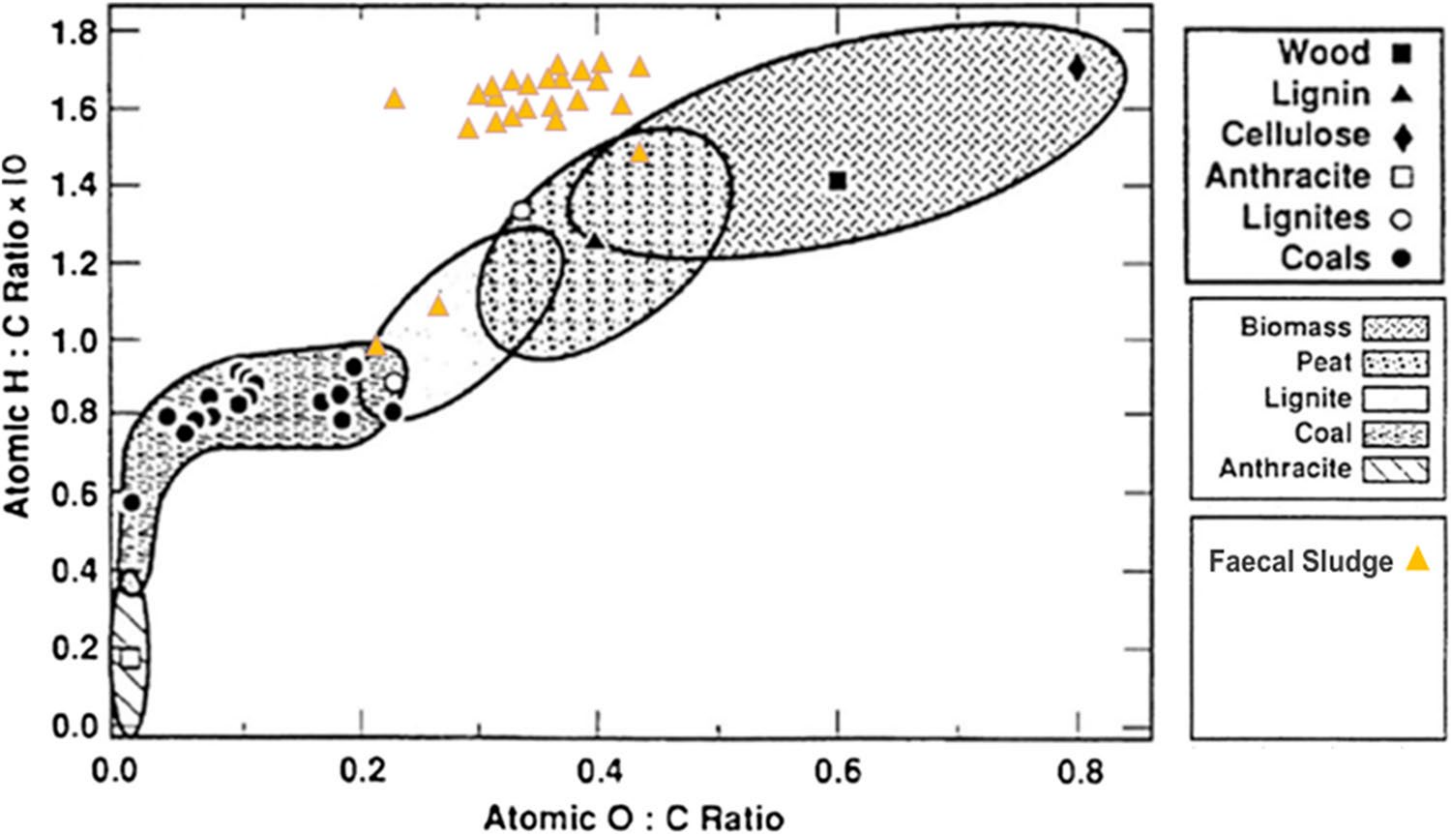
Position of FS in van Krevelen diagram adopted from Baxter [41]

Furthermore, the molar ratios of hydrogen and oxygen to carbon were enumerated for the FS samples and were plotted in a *van Krevelen diagram* [40]; see Fig. 4 to better understand the position of FS as solid fuel with respect to traditional fuels and biomass. The better the fuel, the closer it is to 0 value. The H:C and O:C for the FS samples were in the range of 0.25–0.50 and 0.96–1.70. As illustrated in Fig. 4, the FS samples possess high O:C and H:C ratios compared to coal and lignite. Most samples lie above the boundary of peat and biomass and a few of them lie within the boundary of lignite. In addition, the ratios are not close to 0, which in terms represents the suitability of solid fuel to that of fossil coal. Thus, FS could be seen here as less suitable as compared to biomass.

The N concentration varied between 2.5 to 4.5% DM with an exception of Pit_2 and Pit_5, where N concentration was even exceeding 5% DM, in agreement with [11] who measured N concentrations ranging between 1.5 to 4.3% DM for VIPs and UDDTs, and around 3.2% DM for FS collected from drying beds [5]. One should note, in case of lignocellulosic biomass, in *general*, the N content is remarkably lower, i.e. it does not exceed over 1% [38, 39]. Such high concentration of N is problematic during combustion leading to the formation of toxic gases like NO_x_ and N_2_O [38, 40].

The general S concentration was in between 0.2 to 0.7% DM, with few exceptions where a higher quantity of S was present in Pit_4T, Pit_5T, UDDT_1T, UDDT_2T and VIP_3T between 0.8 to 0.9% DM. Large differences in the top might be due to biological degradation and subsequent release of H_2_S in bottom section that tends to go up, which is pH dependent [5]. Somorin and Kolios [13] also explained the retention of S in FS under the presence of high calcium oxide concentrations in sludge. When inorganic elements in sludge react with S and O, large complex agglomerates are formed, contributing to corrosion, fouling, slagging and erosion. Similar results were also reported by Gold and Ddiba [5], Ward and Gold [16] and Hafford and Ward [20] with a S content of 0.7±0.1% DM to 1.7±0.1% DM in FS collected from a vacuum truck. Thus, even with the variability, the S content from FS in study is in low concentration which on a brighter side will form low amounts of SO_x_ like that of lignocellulosic biomass [40].

### Higher heating value

**Fig. 5:**
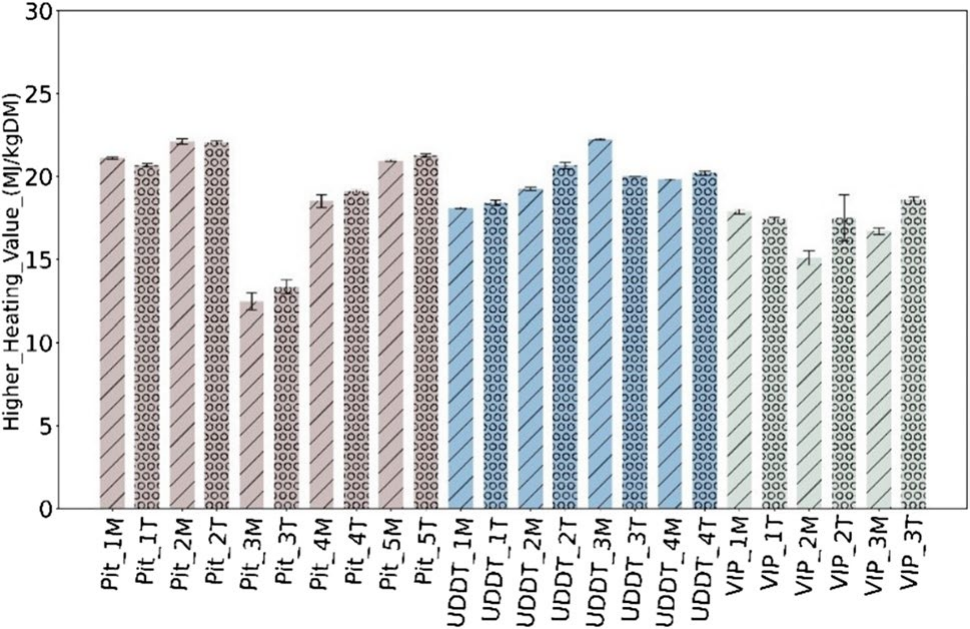
HHV of FS in MJ/kg DM

As shown in Fig. 5, HHV expressed on dry basis is quite variable throughout OSS containments, specifically within the pit latrines with a range between 12 to 22 MJ/kg DM. The fluctuations were notably lower in VIPs and UDDTs with values between 15–20 MJ/kg DM and 18–22 MJ/kg DM respectively. FS from UDDTs showed less variable range than from VIPs and pit latrines, similar to what was observed for the ash content as shown in Section 3.1. Note, no trend or relation could be established between HHV and depth of containment and type/source of the FS. Such variability is in agreement with prior researches concerning the fuel potential of FS [12, 20, 41–44]. However, the values in the present study are higher compared to other researches giving a range between 4 to 18 MJ/kg DM [5, 11, 16, 44] and are close to those of lignocellulosic biomass, typically around 16 to 19 MJ/kg DM [19]. The addition of wood chips and/or oil may explain those results [13].

Based on prior studies, during this research, theoretically FS can be broadly classified into two categories with respect to HHV (i) low-grade FS, where HHV is lower than 15 MJ/kg DM, and (ii) high-grade FS, where HHV is greater than 15 MJ/kg DM. In this research, only the sample from pit latrine 3 falls into the low-grade category.

However, it is important to mention the sample preparation technique adopted. During this research non-faecal particles like stones, gravels (which will decrease the overall HHV of FS) and fibre, plastic and paper (which will increase the overall HHV of FS) were removed by sampling team as well as during the sub-sampling recommended by [6, 12] (as explained in Section 2.1). This may have an impact on HHV at the end—or not—as some particles removed decrease HHV and some increase it.

**Fig. 6:**
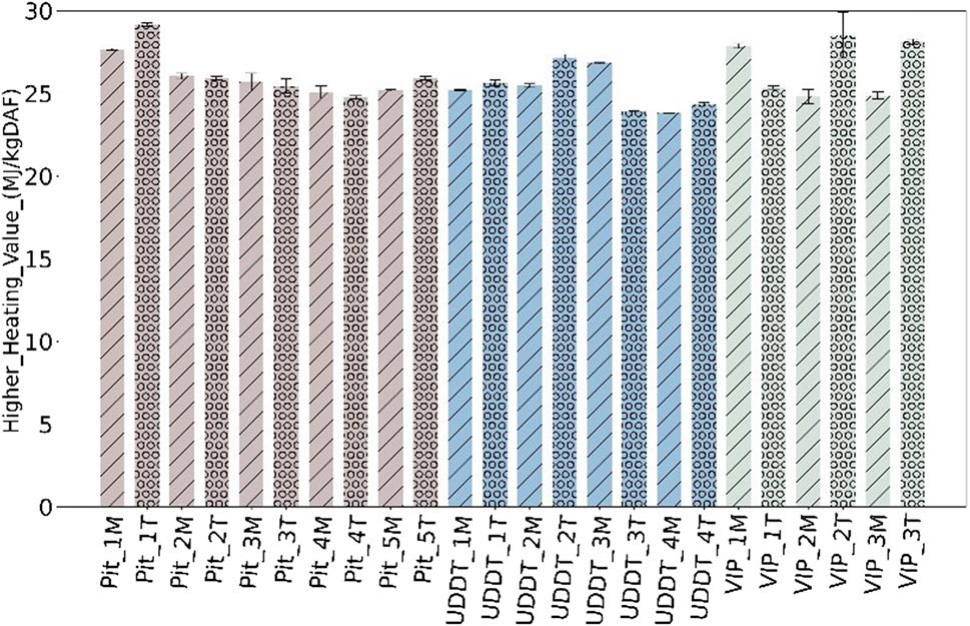
HHV of FS in MJ/kg DAF

To understand the reason behind such variability, the HHV and organic composition were represented on dry-ash free basis (DAF) (Fig. 6), which had not been done in FS studies previously. Interestingly, the range of HHV on a dry ash-free basis is between 24 29 MJ/kg DAF, quite similar with a lower fluctuation across and within each containment. The heterogeneity shown in Fig. 5 can therefore be attributed to the difference in ash content, which is itself attributed to external factors, rather than the intrinsic organic composition of FS.

**Fig. 7:**
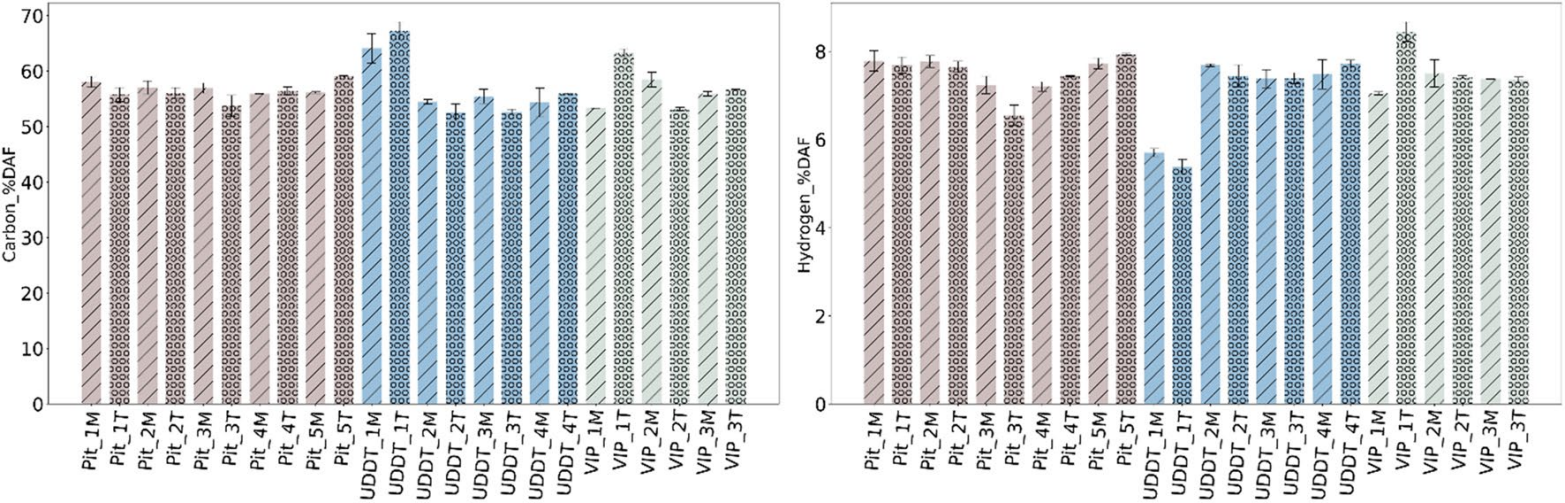
C and H content in % DAF

However, while representing the organic composition of FS in terms of DAF, the fluctuations smoothened between the depth and containment systems as delineated in Fig. 7, where C content varied between 53 to 63% DAF, while H, N and S showed smaller variation between 7–8% DAF, 4–7% DAF and ≤ 1% DAF respectively, which again indicated towards the variability in inorganic composition as discussed in Section 3.4.

### Inorganic elemental composition

#### Major elements

**Fig. 8:**
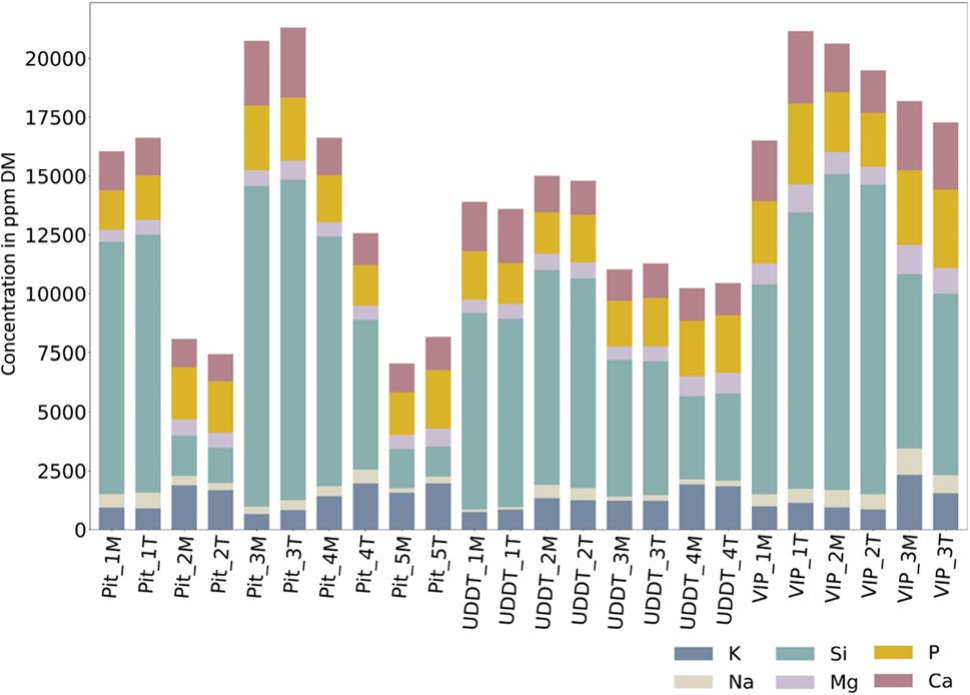
Major inorganic elemental composition of FS in ppm DM

Despite the variability within FS inorganic concentration presented in Fig. 8, it is interesting to note that all FS showed the same major inorganic elements in the same order: Si (1277–13340 ppm DM) followed by P (1664–3341 ppm DM), Ca (1136–2967 ppm DM) and K (658–2327 ppm DM) and smaller much proportions of Mg (521–1250 ppm DM) and Na (117–1117ppm DM). Similarly, with other properties, no trend could be derived based on the depth of containment for the aforementioned elements. Although Krueger and Fowler [11] did not report Na concentration, they measured similar concentrations of major elements Si, P, Ca, Mg and K among others.

Such high concentrations of Si in FS specifically from pit latrines and VIP latrines can be attributed to the fact that none of the pits or VIP latrines were lined causing the soil percolation in FS at the time of collection, as the samples were directly collected from the containment and not at the time of emptying or discharge as done for [5, 11, 14]. This emphasizes the need of standardized protocol for FS collection and analysis [6, 12].

Based on the information gathered on the food consumption behaviour, the most commonly consumed foods were ugali (maize), kale, rice and githeri (a Kenyan traditional meal of maize, beans and legumes). These foods are known to be rich source of P, K, Mg, Ca, Cl, Mn, Na and Fe intake [45]. The concentration variability of major elements could be referred to the diet of the community.

Interestingly, FS samples have quite similar composition in major elements as lignocellulosic biomass, which contains essentially K, Si (mainly in agricultural residues), Ca (mainly in woody biomass) and Si (mainly in grasses), and to a lower extent Mg and P, and less commonly Na [38, 39, 46]. In literature, several authors described lignocellulosic biomass to be quite heterogenous with respect to inorganic composition [38, 39]. Kim and Kang [47] and Hafford and Ward [20] pointed out that the presence of high quantities of Na, K and other alkali elements results in corrosion and fouling during combustion. This can be indication towards FS not being suitable combustion feedstock alone and should be used in co-combustion with lignocellulosic biomass. Thus, more analysis could indicate towards compete understanding of nature of combustion like lignocellulosic biomass.

#### Minor elements

**Fig. 9:**
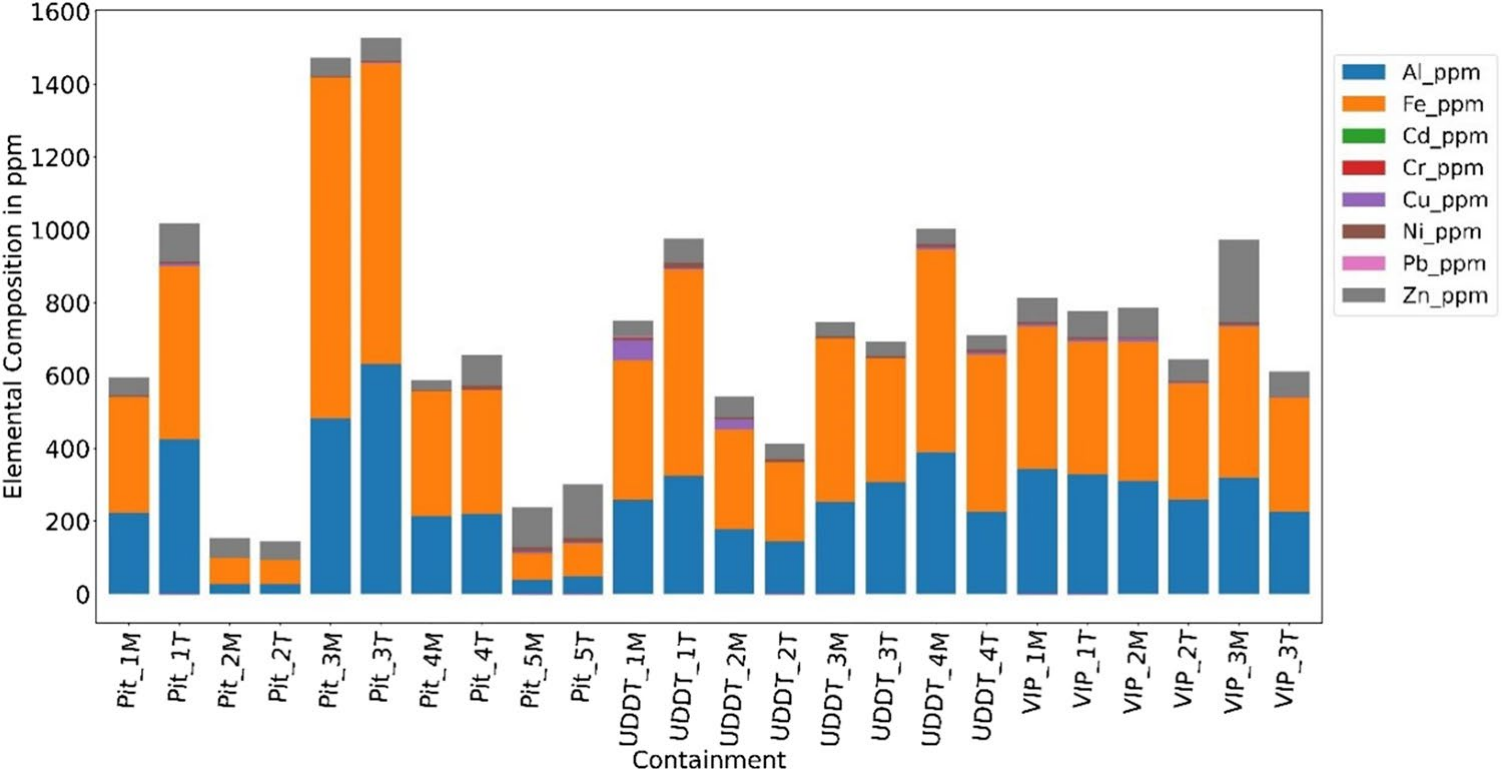
Minor element concentration of FS samples in ppm DM

Figure 9 depicts minor elements, where the concentration is dominated by Fe, Al and Zn with overall concentration between 66–936 ppm DM, 30–630 ppm DM and 30–230 ppm DM respectively. Such high concentration in these selected trace elements can be broadly attributed to the fact that humans are exposed to Al and Fe via dietary components and external pathways like cooking in Al and cast iron utensils, food processing and packaging which ends up in the diet, or via skin contact and inhalation through nose, of which most is excreted out of the body [48, 49]. Other minor elements were present in traces like Cu (< 50 ppm), Ba (< 5 ppm), Sr (< 2 ppm), Cr (< 1ppm), Ni (< 12 ppm) and Pb (< 1 ppm). Lastly, Co and Cd were present just about close to the detection limit.

The origin of heavy metal concentration in FS is, besides presence in excreta, through industrial effluents, leachate infiltration and disposal of solid waste into the containments. The latter was also observed by [43], as the common waste disposed of in the OSS containments was polyether, paper, menstrual hygiene products, glass bottles, batteries, among others, as reported by the sampling team (also discussed in Section 3.1). It should be kept under serious considerations that even though present in trace quantities, heavy metals are present in the residual ash and the amounts tend to accumulate after combustion [2, 11, 50]. As discussed by Huron and Oukala [9], legislations usually refer to gas emissions after combustion, but some specifications can be found on the quality required for solid biofuel, more specifically wood, entering combustion units (generally <1000ppm). To the author’s knowledge, such specifications do not exist for FS yet. Even though there is no limit on FS, the total minor element concentration for most of the FS is still below 1000 ppm DM which is acceptable.

## Conclusions and recommendations

After an extensive analysis of the combustion properties of twenty-four FS samples collected from three different OSS pit latrine, VIP latrines and UDDTs at two depths, the following conclusions and recommendations could be drawn:

- Heterogeneity observed in terms of HHV within and across the containments were found to be largely corelated to the variability in the ash content—from 15 to 50% DM, while the C, H and O composition of FS was found similar in all samples.
- While ash content varied significantly among samples and different containments, the main inorganic elements forming this ash were found to be always in the same order, i.e. Si **˃** P **˃** Ca **˃** K, followed by Mg and Na. Thus, the variability associated with FS is largely due to varying ash content, not ash composition. Furthermore, it is evident that sanitation technology greatly influences the ash content and consecutively the HHV. The FS from UDDTs appears to be most suitable and showed consistency when it comes to use it as a fuel.
- HHV was in a higher range of values compared with literature, between 13 to 22 MJ/kg DM. Such high values of HHV together with low S content (<1% DM) and overall minor element concentration (<1000 ppm DM) could be promising for its use as solid fuel. It should be taken with caution considering the systematic bias potentially induced by sample collection and preparation according to the existing guidelines on FS characterization.
- The high ash content and high concentration in Si, P, K and Ca, as well as high N content, imply that FS should *not* be seen as feedstock for combustion alone but as feedstock for co-combustion together with lignocellulosic biomass. Subsequent investigations may consider co-processing with lignocellulosic biomass as area of interest, as it may hold the potential to yield significant benefits when tackling various waste.
- The study discussed three types of sanitation systems from one city, maintaining the consistency in methodology of sampling and analysis. It is recommended for future studies to keep adopting such standard protocol and apply the same methodology to other sanitation systems/cities, so that a consistent database on FS can be progressively built and made available to the community of researchers and practitioners.
- Finally, on a broader viewpoint, it is also advised to deal in future studies with related questions of sanitation practices, including the design or retrofitting of current systems, and behaviour change, including the prevention of solid waste entrance in containment.
